# Preferences, predictions and patient enablement: a preliminary study

**DOI:** 10.1186/1471-2296-14-116

**Published:** 2013-08-14

**Authors:** Carl J Brusse, Laurann E Yen

**Affiliations:** 1 Australian Primary Health Care Research Institute, The Australian National University, Building 63, corner of Mills & Eggleston Roads, Acton 0200 ACT, Australia

## Abstract

**Background:**

The widely used patient enablement instrument (PEI) is sometimes contrasted against measures of patient satisfaction as being a more objective measure of consultation quality, in that it is less likely to be positively influenced by fulfilling pre-existing expectations for specific consultation outcomes (such as prescriptions or referrals). However the relationship between expectation and enablement is underexplored, as is the relationship between ‘expectation’ understood as a patient preference for outcome, and patient prediction of outcome. The aims of the study are to 1) assess the feasibility of measuring the relationship between expectation fulfilment and patient enablement, and 2) measure the difference (if any) between expectation understood as preference, and expectation understood as prediction.

**Methods:**

A questionnaire study was carried out on 67 patients attending three General Practices in the Australian Capital Territory. Patient preferences and predictions for a range of possible outcomes were recorded prior to the consultation. PEI and the actual outcomes of the consultation were recorded at the conclusion of the consultation. Data analysis compared expectation fulfilment as concordance between the preferred, predicted, and actual outcomes, with the PEI as a dependant variable.

**Results:**

No statistically significant relationship was found between either preference-outcome concordance and PEI, or prediction-outcome concordance. Statistically insignificant trends in both cases ran counter to expectations; i.e. with PEI (weakly) positively correlated with greater discordance. The degree of concordance between preferred outcomes and predicted outcomes was less than the concordance between either preferred outcomes and actual outcomes, or predicted outcomes and actual outcomes.

**Conclusions:**

The relationship between expectation fulfilment and enablement remains uncertain, whether expectation is measured as stated preferences for specific outcomes, or the predictions made regarding receiving such outcomes. However the lack of agreement between these two senses of ‘patient expectation’ suggests that explicitly demarcating these concepts during study design is strongly advisable.

## Background

### Satisfaction and enablement

One dimension by which health systems differ from each other is the degree to which they treat health care as a public good to be delivered, as opposed to a consumer-demanded commodity to be supplied in a health care marketplace. In private or mixed systems where patient choice and the ability to ‘shop’ for healthcare as a service commodity plays a significant role, the use of patient satisfaction measures makes intuitive sense. In this regard, it is not surprising that securing patient satisfaction is increasingly seen as a primary performance measure for clinicians, especially in the United States [1]. Furthermore, to the extent that consultations are seen as service commodities (with patients seen as active seekers and consumers of those services) then it is also intuitively plausible that satisfaction might depend on the fulfilment of prior expectations for medications, tests, referrals, and other onward services for which the general practitioner is in effect a ‘gatekeeper’.

Perhaps for these reasons (and for the potential health economic effects), there has been considerable research interest in testing the link between fulfilment of specific patient expectations for such ‘service outcomes’ and resultant satisfaction. Fulfilling an expectation for a desired medication appears to be significant in some studies set within specific contexts [1] though not in others [2]; similar results can be found regarding expectations for tests or referrals. A literature review by Rao et al. in 2000 concluded that, while fulfilment of expectations for a diagnosis or good doctor-patient communication were more significant, there is indeed a modest relationship between the fulfilment of service outcome-expectations and satisfaction [3]. This suggests that specific pre-consultation outcome-expectations (as opposed to more abstract expectations for diagnosis, empowerment, or clinician communication/empathy) can influence a patient’s self-reported satisfaction with a consultation.

With the wide variety of health issues that present, primary health care is handicapped by a lack of alternative, generally-appropriate, patient-focused performance measures. One attempt at filling this gap is the Patient Enablement Instrument (PEI): an easily administered self-report indicator of consultation quality by patients, devised by researchers in the UK as a “conceptually distinct” alternative to more widely used tools to measure patient satisfaction [4,5]. Whereas patient satisfaction is a broadly economic concept analogous to satisfaction with any other transaction of services, patient enablement surveys specific, clinically-relevant attitudes and phenomena such as improvement in the patient’s understanding of their illness, and in their capacity and confidence with respect to treatment and self-management.

Key to the current discussion is that enablement purports to give a representation of consultation quality that is less influenced by non-clinical factors, for example patient reluctance to express dissatisfaction about clinicians with whom they have an on-going relationship, or any tendency to express dissatisfaction purely on the basis of whether or not prior expectations for referrals (etc.) had been met. As Howie et al. put it: “it is recognized that satisfaction may reflect whether or not expectations have been met rather than whether or not benefit has been achieved. In that case, it is debatable whether satisfaction can truly be regarded as an outcome. For such a measure to be useful it should be centred on issues that patients identify as important to them rather than on issues that doctors believe to be important. We believe that that the Patient Enablement Instrument meets these requirements. It also seems less likely simply to reflect whether expectations have been met.” [5].

The PEI was specifically intended for use in NHS services in the United Kingdom, where elements of it have been incorporated into broader quality indicators for consultations [6] and the GPAQ General Practice assessment questionnaire [7]. In Australia, a variant is encouraged and distributed by the Australian Medicare Locals Alliance as part of the Patient Enablement and Satisfaction Survey (PESS) for the performance assessment of practice nurses [8].

In the context of consultations, the PEI has been demonstrated to be distinct from metrics of patient satisfaction [5], to not vary significantly between casemix types [9], and to deliver similar results in various different territories and in non-English translation [10-15]. Importantly, it has been shown to be positively correlated with higher assessments of clinician empathy and/or communication skills [16-18], longer consultation times [9,14] and continuity of care [9,14,18], with one study also finding higher PEI scores to be predictive of better health outcomes after one month [16]. Enablement also varies with some non-clinical variables, with significantly lower PEI scores seen among middle-aged patients and significantly higher scores in English-speaking countries among patients who speak another language in the home, which may or may not be explained by systematic differences in self-report behaviour or significant differences in expectation [18].

### Rationale: expectations and outcomes

The originators of the PEI instrument saw prior preference for ‘service outcomes’ as having a potential spoiling effect when using satisfaction measures as outcome measures: “Although satisfaction questionnaires generally attempt to reflect patients’ perceptions of outcome, their structure often appears to measure the extent to which expectations relating to the process of delivery of care have been met, rather than whether there has been any achievement of specific health gain” [5]. The distinctiveness between satisfaction and enablement observed in that same study implies that patients can be enabled but not satisfied, by (for example) being less than fully satisfied by outcomes of a consultation (which didn’t fulfil their expectations), yet still benefited by it as captured by enablement. The most plausible mechanism to account for this distinctiveness is the established sensitivity of the PEI instrument to quality-correlated features of the consultation as discussed, e.g. clinician empathy/communication, consultation length and continuity of care.

If so, enablement would be less likely to track fulfilment of prior ‘service outcome’ expectations and more likely to track relevant features of consultation quality, and would offer a means of assessing the quality of a consultation that stands separate from a ‘demand & supply’ model of service provision.

### Rationale: uncertainty and ambiguity

However there is a gap in the literature that hinders making any strong conclusions about the robustness of enablement measures with respect to expectation fulfilment (for ‘service outcomes’ as discussed here), and thereby threatens to undermine the rationale for enablement as described above. The relationship between expectation fulfilment and enablement has not been given the same research attention as that between expectation fulfilment and satisfaction. While it is clear that enablement is an outcome that goes beyond the mere provision of healthcare items, (and the ideal of enablement better tracking specific health gain has a certain narrative logic) it is not clear how sensitive it is to those prior expectations in the first place. In other words, it has not been established if it is in any way immune to the spoiling effects of those particular influences.

A secondary, methodological concern here has to do with the terminological ambiguity of ‘expectation’. The notion of an expectation – if not carefully phrased – can be ambiguous between desire and prediction: i.e. between what a patient *desires* from the clinician and what a patient *predicts* the clinician would do. As Williams et al. put it: “a patient can have ‘expectations’ about how a doctor will behave yet not necessarily desire what is expected” [19].

Simply surveying patients with a single question about their expectations with respect to prescriptions, tests and referrals may conflate these two senses of expectation, and therefore not necessarily isolate the intended microeconomic phenomenon: which outcomes (if any) the patient desires or prefers as he or she enters the consultation.

### Preliminary study

This preliminary study was conducted to examine the feasibility of measuring the relationships between enablement, as measured by the PEI; and the fulfilment of two distinct senses of outcome expectation.

More specifically, we aimed to measure whether enablement varies according to concordance or disagreement between any of the following:

1. A patient’s prediction of what their GP would recommend or prescribe with respect to care, such as pharmacy scripts, tests, referrals and self-management (outcome prediction)

2. A patient’s own opinions/preferences for such care outcomes (outcome preference),

3. The care outcomes that the GP actually recommended (actual outcome).

The hypothesis tested is that patient enablement will be positively correlated with the concordance between the patient’s outcome preferences and actual outcomes, and *not* (or less) positively correlated with the concordance between the patient’s outcome predictions and actual outcomes.

## Methods

We designed a survey for a preliminary study with patients at three local general practices.

Given the narrow scope of this study (to test the relationship between PEI and outcome preference/outcome prediction) we developed a list of possible consultation outcomes, using medical and nursing clinicians to identify and classify common outcome types. The sixteen items on these lists were phrased so as to represent persons and professions that might be recommended to become involved in the patient’s care as a result of the consultation (i.e. further ‘services’ to be recommended or permitted by the GP), with brief explanatory text. For this study, the list was not further validated by methods such as video trials or patient involvement. The full list of outcome options was:

• The doctor (GP) seen today (e.g. for a follow-up consultation)

• Other doctor/GP(s)

• Specialist doctor(s) (e.g. surgeon, dermatologist)

• Psychiatrist

• Other mental health worker(s) (e.g. psychologist, social worker, counsellor)

• Other health professional (e.g. nurse, physiotherapist, audiologist, optometrist)

• Hospital staff for admission to hospital

• Pharmacist/Chemist (e.g. for medications)

• Laboratory testing staff (e.g. for blood tests or x-rays)

• Complementary health practitioner (e.g. naturopath, massage therapist)

• A personal helper (e.g. partner, spouse, relative, friend, home help)

• Self-help organisation (e.g. arthritis, diabetes or depression support group)

• I am not certain

• No-one

• manage the condition myself (self-help)

• Other

The questionnaire itself was designed in two parts: a pre-consultation form and post-consultation form. The pre-consultation form surveyed basic demographic data and broad clinical information, including how well the patient knew the doctor they were seeing (using a five point scale) and the patient’s reasons for attending the consultation (e.g. ‘new physical problem’, ‘on-going physical problem’). This information was intended to allow for validation of PEI results by comparison against existing studies, to inform the future design of any larger study, and to provide a basis to compare patient responses according to caseload type. The above outcome list was presented to the patient twice: once for the participant to *predict* the outcomes of the consultation with their doctor, and a second time to express their *preferred* outcomes.

The post-consultation form presented the list a third time, but in this case for the GP to select the actual outcomes of the consultation. On the reverse side of this form was the PEI instrument, to be filled out by the patient immediately after the consultation. We used the PEI as originally designed [4]: six questions with four possible responses scored as zero (“same or less”, or “Not Applicable”), one (better/more) or two (much more/better), with a per-patient PEI score being the sum of scored responses. This results in a scale from zero to twelve, with an expected linear distribution (as opposed to satisfaction measures, which are typically positively skewed by patient reluctance to express dissatisfaction with individual clinicians [5,13,20]).

The study was conducted over one month at three metropolitan practices from the practice-based research network (known as PracNet) affiliated with the Australian National University Medical School. Consecutive available patients in practice waiting rooms were approached by a research nurse, invited to participate in the study, and supplied with an information sheet and a consent form. All patients over the age of 18 were invited to participate; participants were not randomised or otherwise selected, however some patients declined to participate or were ‘missed’ due to time constraints.

Researchers presented participants with the pre-consultation form and were available to assist in filling it out, if needed, in the waiting room prior to the consultation. The pre-consultation form was retained by the patient during the consultation in an opaque envelope. At the conclusion of the consultation the GP filled out their side of the post-consultation form and passed it to the patient, who left the consultation, filled in the PEI section, and sealed both forms in the provided envelope for return to the researchers or practice reception. No identifying information was gathered and at no point did the GP or any practice staff see any of the patient’s responses. Additional information was gathered about participating GPs, so that professional development credit could be recorded.

Ethics approval for the study protocol was obtained from the Australian National University’s Human Research Ethics Committee (2011/479).

Analysis was carried out with SPSS 19 software. For the purposes of this study, the concordance between outcome preference, outcome prediction and actual consultation outcome was measured by counting the disagreements between items on the option lists as filled out by patient and GP, as a number between zero and either sixteen (with all options considered) or fourteen (with the non-specific options “uncertain” and “other” excluded). Disagreement counts in each case were compared against the PEI scores, and further comparisons were made between the mean PEI scores of patients whose expectations were or were not met for specific outcome types.

## Results

Sixty eight participants agreed to participate in this preliminary study out of seventy four approached, and for sixty two participants all required information was gathered on preferred, predicted and actual outcomes, and a valid PEI score obtained.

The PEI scores showed a linear distribution with an overall mean of 4.31, and the relationship between PEI score and how well the patient knew the doctor was a strong and statistically significant. These results are typical of PEI characteristics from large-scale studies [5].

The discordance between outcome preference and actual outcome (the number of disagreements) was between one and six with a mean of 2.9. The Pearson Correlation coefficient with PEI was 0.164 and statistically insignificant (0.204, two-tailed). Treated as six discrete groups according to disagreement count, there were no significant differences between the PEI means of these groups. Divided into two groups with disagreements less than three (N = 21) and greater or equal to three (N = 41), there was no significant difference between PEI means (t(60) = 0.762, p = 0.449).

Discordance between outcome prediction and actual outcome was also between one and six, with a mean of 3.2. The Pearson Correlation coefficient with PEI was 0.189 and statistically insignificant (0.141, two-tailed). Treated as six discrete groups according to disagreement count, there were no significant differences between the PEI means of these groups. Divided into two groups with disagreements less than three (N = 22) and greater or equal to three (N = 40), there was no significant difference between PEI means (t(60) = −0.162, p = 0.872).

The sample size and wide variance in PEI scores created margins of error such that no conclusions can be made regarding differences between the means. The 95% confidence interval for the entire population’s PEI mean was between 3.34 and 5.22; easily containing the PEI means for the different subsets of patients we used for comparison.

A third discordance test was carried out between outcome prediction and outcome preference. Disagreement here was between one and nine, with a mean of 4.2. The Pearson Correlation coefficient with PEI was 0.184 and statistically insignificant (0.152, two-tailed). There were similarly no significant differences between the PEI means when divided into discrete groups according to disagreement.

## Discussion

### Expectation, enablement, and quality

The results of this preliminary suggest that there is indeed no positive correlation between the fulfilment of patient expectation and their enablement. If anything, the weak and statistically insignificant trend in the data was inclined in the opposite direction: with the average enablement score increasing with increasing number of expectation-outcome disagreements (for both senses of expectation). Though not statistically significant, this is in itself an interesting result and should be discouraging for any ambition to uncover a positive relationship between enablement and expectation akin to the mild one previously observed between satisfaction and expectation.

It is also worthy of note that explicitly separating outcome preference and outcome prediction appears to be validated by the results of this study: as the discordance between these two senses of expectation was greater than the discordance between either of them and actual outcome.

On consideration, a negative correlation between expectation-outcome concordance and enablement might even be expected given the nature of the enablement measure. Among its other questions, the PEI asks the patient to rate how *improved* their understanding of their illness is. Should a patient find their preconceptions substantially corrected by the GP during the consultation, their self-assessed improvement in understanding would likely be greater than it would by simply having those preconceptions reinforced.

However this highlights an interesting feature of the PEI as a measure of performance: the comparative language of the PEI questions mean that it scores for *improvements* in patient understanding and capacity (and so forth) rather than any absolute level of understanding and capacity. A high PEI therefore requires a patient who was previously in a ‘poorly enabled’ state, for whom their understanding and capacity with respect to their illness has room to improve. This might be expected to lowly rate those consultations (regardless of quality) where patients already possess a good level of understanding and capacity.

A potentially worrying (albeit speculative) example of such ‘false negatives’ might be the following: a well-conducted chronic care management consultation as part of an on-going and well-constructed care plan, with a (by now) well-informed and confident patient, during which nothing surprising is discussed. If we assume that the patient answers PEI questions literally, then they should answer each enablement question as “same or less”; in other words giving an overall PEI score of zero.

This scenario is merely a thought experiment, and we do not mean to imply that enough actual consultations resemble this enough to cause any real concern about the applicability of the PEI in the real world (this would take empirical investigation). What it serves to illustrate is that while enablement (as measured by the PEI) appears to be a better measure of consultation benefit than satisfaction measures, like all measures there will be cases where it may not track what we had intended, or assumed. Philosophically speaking, we might interpret this case as one where *benefit* (as tracked by PEI) comes apart from *quality*, and perhaps also from long term *value*. This is plausible: a regular consultation (as part of an on-going continuity of care) can be more valuable in terms of the future health of the patient than the immediate benefits of it would suggest. Conceptual clarity in the measurement of health outcomes, as well as what the goals of those outcomes should be and how they should be incentivised, is not a simple matter.

### Study limitations

There were several limitations to this preliminary study which should be acknowledged, and there are recommendations to be made for further studies along these lines with regard to study design. In our study, we asked GPs to indicate which of the care options were made during the consultation, from a list of options. Though it helped to engage GPs in this study, relying solely on this feedback as the record of outcome relied on the patient and doctor reading and understanding our taxonomy of health care providers in the same manner. It therefore would have been better to ask patients to record the care outcomes as well. In this respect, further validation of any common list of ‘service outcomes’ should involve observed involvement/trials of patients and clinicians, as well as explicit reference to the contrast class of other outcomes and features of the consultation (for example as isolated in empathy studies). The lack of such development is a weakness of the current study.

There are also inherent weaknesses in the waiting-room model of patient recruitment and questionnaire delivery. While the participation rate was encouragingly high, non-participation was biased toward non-English speaking patients and patients who presented in the waiting room too close to their eventual appointment time. Samples are necessarily internally non-randomised cluster samples, and clusters themselves (defined by practice and collection time-frame) must therefore be carefully selected and randomised to ensure a more representative sample.

We must also reiterate that this preliminary study, with its high number of possible responses, is too small to make any strong conclusions. PEI averages are known to vary widely between different clinicians (reflecting communication and empathy characteristics), and insufficient data were collected to characterise and individuate the effects of clinician difference in the data set. Likewise, a larger study would be able to determine if length of consultation were correlated with likelihood of expectations satisfied, which may have explanatory effect here (no significant correlation between consultation length and PEI was observed in our sample).

Finally, given the lack of any satisfaction measures used in this study, no direct contrasts between satisfaction and enablement should be made. This would be an obvious addition for any full-scale study.

### Recommendations for further research

With our study, we compared mean PEI scores for patients who did or did not report an on-going physical health problem, new physical health problem, on-going mental health problem or new mental health problem. Though no statistically significant differences were found, we did not record the exact nature of the consultation and the issues addressed. Larger, redesigned versions of the current study might therefore be recommended to better investigate the validity of PEI in this regard, comparing the effects of improvement-based vs. improvement-free question language on responses between specific consultation caseload types, contrasted against satisfaction measures.

We can recommend that the intended sense of expectation (preference or prediction) be made explicit in the wording of any questions about patient expectations, because while neither quantity was found to be interestingly correlated to enablement, patients demonstrated a clear differentiation between the two concepts through their survey responses. This phenomenon may deserve more attention in itself.

We believe that our technique of isolating what we have called ‘service outcomes’ from other outcome considerations could serve a potentially valuable role in future patient expectation studies, though (again) further validation would be required.

Part of the attraction of enablement as a measure of quality is as a more clinically ‘objective’ counterweight to consumer/patient satisfaction – a valuable tool in the absence of more specific metrics for primary health care. However the nature of enablement itself has not been fully addressed in the published literature, and more disseminated research is called for before enablement is broadly relied upon for making informed decisions in policy and practice. One alternative strategy, which we suggest for further consideration, would be benefit and quality measures tailored to presentation, caseload type, and context. Early research on the PEI included investigation of such variation [4] and further research along these lines might be recommended for specific health service contexts.

Indeed, with the on-going reform, development and diversification of the primary health care sector (especially in Australia) we believe there may be new scope for caseload-specific classification and assessment of services performed by primary health care professionals in general, be they delivered via the traditional GP consultation format or otherwise.

## Conclusions

When framing and motivating the study hypothesis we briefly discussed the economic intuition of the consultation being a ‘gateway for consumer outcomes’ – a logical outcome of seeing the patient as self-directed consumer of health care services with a gatekeeper model of primary health care as a front-line ‘referral service’ – an intermediary between consumers and other desired products. These are not merely abstract concepts: the model is only valid if patients behave as consumers in this regard, and the degree to which they do so should be reflected in the relationship between expectation-fulfilment and satisfaction (and possibly enablement). The lukewarm relationship between meeting pre-consultation expectations and satisfaction in the literature already casts doubt on this. Furthermore if the relationship between expectation fulfilment and *enablement* does indeed go in the other direction, then (uncertainty about the exact nature of enablement measures notwithstanding) this would support the idea of enablement as a measure ‘uncorrupted’ by preferences for service outcomes, but may also feed into interesting considerations when deciding what constitutes quality in health care.

Our small study by no means tested these assumptions adequately, but it points to a way in which they might be tested, and this is potentially significant. Enablement and/or other ‘objective’ measures of health care quality (which align with policy priorities over and above merely satisfying the patient qua consumer) should be more closely examined for use in the primary health care setting.

## Competing interests

The authors declare that they have no competing interests.

## Authors’ contributions

CB participated in the conception and design of the study, carried out the statistical analysis, and drafted the manuscript. LY participated in the conception and design of the study, helped to draft the manuscript and provided critical revision. Both authors read and approved the final manuscript.

## Pre-publication history

The pre-publication history for this paper can be accessed here:

http://www.biomedcentral.com/1471-2296/14/116/prepub
